# Magnetic segregation effect in liquid crystals doped with carbon nanotubes

**DOI:** 10.3762/bjnano.10.145

**Published:** 2019-07-22

**Authors:** Danil A Petrov, Pavel K Skokov, Alexander N Zakhlevnykh, Dmitriy V Makarov

**Affiliations:** 1 Physics of Phase Transitions Department, Perm State University, 614990 Perm, Russia; 2 Institute of Continuous Media Mechanics, Russian Academy of Sciences, Ural Branch, Perm, 614013, Russia

**Keywords:** carbon nanotubes, liquid crystal, magnetic field, orientational transitions, segregation effect

## Abstract

We study the orientational transitions in a suspension of carbon nanotubes in a nematic liquid crystal induced by an external magnetic field. The case of a finite orientational anchoring of liquid crystal molecules at the surface of doped carbon nanotubes is considered. It is shown that in a magnetic field the initial homogeneous planar texture of the liquid crystal–carbon nanotubes mixture is disturbed in a threshold manner (Fréedericksz transition). The orientational and concentration distributions of the suspension are studied for different values of the magnetic field strength and segregation intensity of the impurity subsystem. The optical phase lag between ordinary and extraordinary rays of light transmitted through a layer of a liquid crystal composite is calculated. The possibility of changing the nature of the Fréedericksz transition from second order to first order is shown. This tricritical behavior is related to the redistribution of the carbon nanotubes (segregation effect) inside the layer.

## Introduction

Composites of liquid crystals (LCs) and nanoparticles are actively studied systems in soft condensed matter physics, since they successfully combine fluidity and orientational order with specific properties of impurity particles, such as ferromagnetic, ferroelectric, metallic or dielectric impurities [1–10]. Adding a small amount of nanoparticles modifies many properties of liquid crystals and leads to the possibility of obtaining new hybrid materials with unique electro- and magneto-optical properties, which opens prospects for new practical applications in optoelectronics, photonics, and display technology [11]. From a physical perspective, these composite materials capable of spontaneous ordering are interesting due to the fact that their properties are determined by the interactions between the embedded nanoparticles and the carrier matrix. The properties of LC mixtures with nanoparticles substantially depend on the material of the particles, their shape, size, and concentration. Anisometric particles are oriented in the LC matrix, which leads to significant changes in the electro- and magneto-optical response of the composite material. Nanoparticles placed in an LC enhance many physical properties, such as susceptibility to external fields [1,11–12]. Thus, the idea of controlling the features of composites by adding a small amount of nanoparticles to an LC matrix is of great interest from a physical point of view.

Carbon nanotubes (CNTs) [13] are a popular material to be embedded in LCs [9,14–17]. Due to a large aspect ratio the physical properties of this carbon nanomaterial vary greatly in different directions. In this sense, the anisotropic properties of CNTs (for example, thermal and electrical conductivities) are attractive for a wide range of applications, including nanoelectronics and optics [2]. A distinctive feature of CNTs is their strong diamagnetism (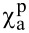 ≈ 10^−5^ to 10^−4^) [18–23]. In the majority of experimental publications [7,16,24–26] the planar type of anchoring between the nanotubes and the LC matrix is noted. For CNT suspensions based on nematic liquid crystals (NLCs) with positive anisotropy of the diamagnetic susceptibility, this leads to a decrease in the critical fields of magnetic [27–28] and electric [14,16,26,29–30] Fréedericksz transition (a threshold process of changing the orientational structure inside an NLC layer under the action of external fields [31]). Experiments show [28,32–34] that in nematic liquid crystal–carbon nanotube (NLC-CNT) mixtures additionally functionalized with ferromagnetic particles, the magneto-optical response increases in comparison with pure LC. Existing theoretical models that describe NLC-CNT suspensions are related to the mean-field theories [35–36], generalizations of the Landau–de Gennes phenomenological theory [37–38] or modifications of the free-energy functional of the LC, which take into account the presence of the CNT impurity [39].

In the present work, on the basis of the free energy functional [39] magnetic-field-induced equilibrium transitions in NLC-CNT suspension have been studied. The influence of segregation effects on the nature of the magnetic Fréedericksz transition and the magneto-optical response of a flat layer of the mixture are analyzed.

The paper is organized as follows. First, we present the continuum theory of NLC-CNT suspension and perform estimations of dimensionless quantities for real mixtures. After that, possible orientational phases of an NLC doped with CNTs are discussed, and the nature of the Fréedericksz transition depending on the segregation intensity of impurity CNTs is analyzed. At the end, we present the results of numerical calculation of the orientation and concentration profiles of the NLC-CNT mixture and its magneto-optical response.

## Theory and Model

**Figure 1:**
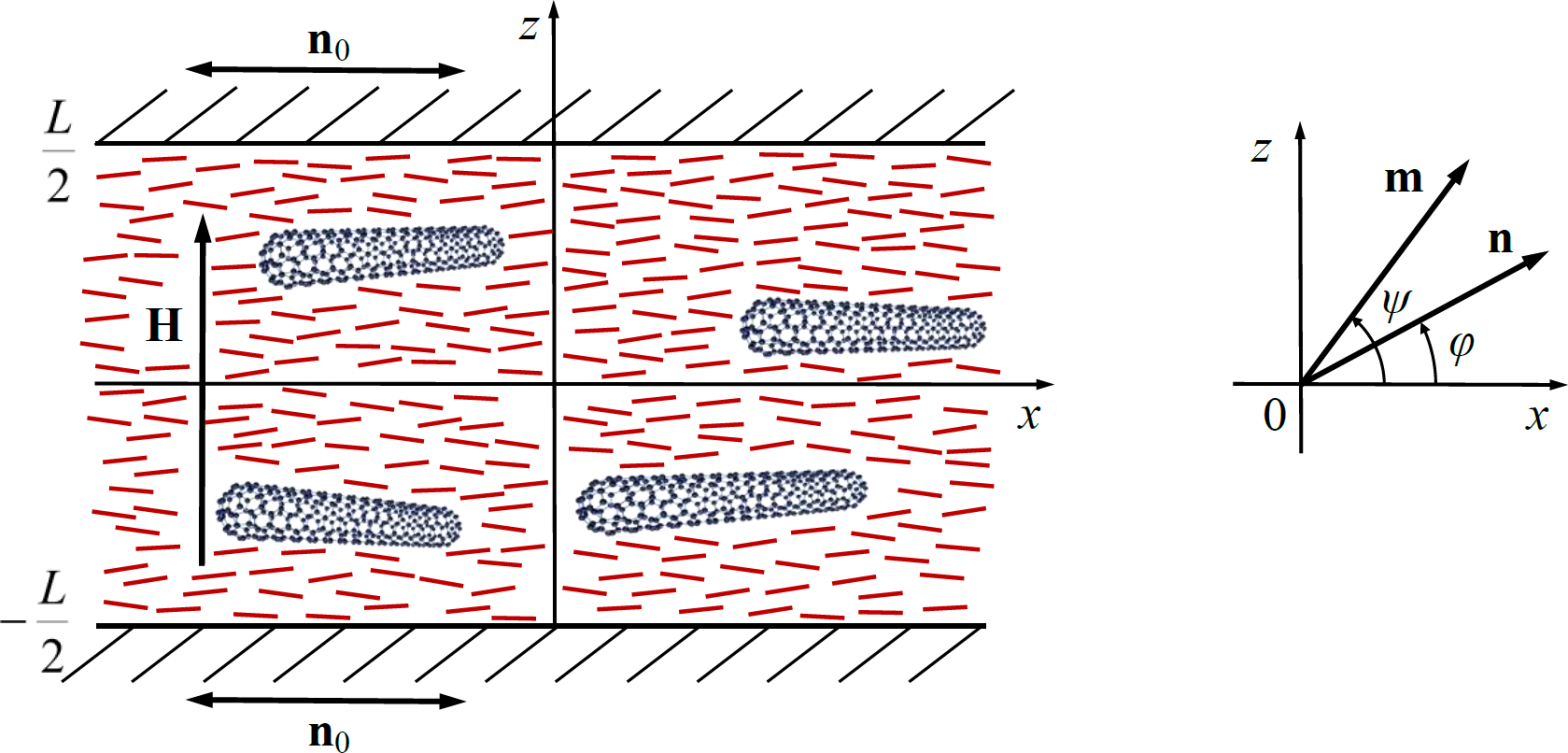
An NLC layer with CNTs in a magnetic field.

Let us consider a low concentration of CNTs suspended in an NLC placed between two plane-parallel plates (Figure 1). In the framework of the continuum theory [39], the directions of the preferred orientation of the NLC molecules and the CNTs are determined using the unit vectors **n** and **m**, respectively. The anchoring of NLC molecules on the surface of the plates will be considered absolutely rigid and planar, so the director of the NLC **n** = **n**_0_ at *z* = ±*L*/2, where the vector **n**_0_ sets the direction of the easy orientation axis on the layer boundaries. The anchoring of the NLC molecules on the surface of CNTs is assumed to be finite and planar, i.e., the directors of the NLC and the CNTs are parallel to each other (**n**∥**m**) in the absence of external fields.

Recently, a continuum theory was proposed in [39] to describe the magneto-orientation response of an NLC–CNT suspension. It is based on the generalization of the free energy of an LC, taking into account the fact that a small amount of CNT impurities has been added to the NLC matrix. The total free energy *F* of such an NLC–CNT suspension in an external magnetic field can be written as follows:

[1]
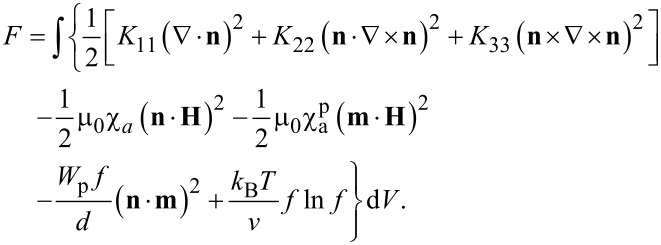


Here *K*_11_, *K*_22_, and *K*_33_ are the Frank constants, **H** is the external magnetic field strength, χ*_a_* *>* 0 and 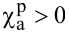 are anisotropies of the diamagnetic susceptibility of the NLC matrix and CNTs, μ_0_ is the magnetic permeability of the vacuum, *W*_p_ is the surface anchoring energy density of the NLC matrix and CNTs, *v* and *d* are the volume and transverse diameter of the CNTs, *f*(**r**) is the local volume fraction of CNTs in the suspension, *k*_B_ is the Boltzmann constant, *T* is the temperature. The first contribution to *F* in Equation 1 is the free energy of elastic deformations of the NLC director field (Oseen–Frank potential). The second and the third summand determine the interaction energy of the NLC and the CNTs with the magnetic field **H**, the fourth summand describes the energy of the interaction of the CNTs with the NLC molecules, and the last term is the contribution of the entropy of mixing an ideal gas of CNTs in the NLC. The volume fraction of CNTs in an NLC sample is assumed to be small (*f* ≪ 1), which makes it possible to neglect the interaction of the carbon nanotubes with each other.

Orientation distortions induced by magnetic field **H** = (0, 0, *H*) are described by spatial distributions of the directors

[2]
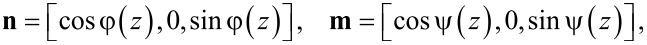


where φ(*z*) and ψ(*z*) are the deviation angles of **n** and **m** from the easy orientation axis **n**_0_ (Figure 1).

The thermodynamic equilibrium state of the suspension is determined from the minimum of the total free energy (Equation 1), which in the geometry under consideration is a functional relative to the angles φ and ψ and the volume fraction *f* of the CNTs. The minimization of *F* (Equation 1) by φ and ψ leads to the following equations of orientational equilibrium:

[3]
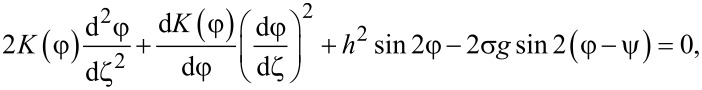


[4]
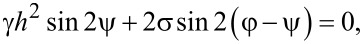


where *K*(φ) = cos^2^φ + *k*sin^2^φ. The minimization of *F* (Equation 1) by *f*, provided that the number of CNTs is constant in the LC layer ∫*f*d*V* = *Nv*, yields

[5]
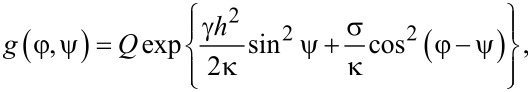


[6]
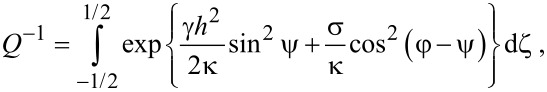


where 
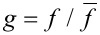
 is the reduced volume fraction of the CNTs in the suspension, 
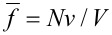
 is the average volume fraction of CNTs, *N* is the number of CNTs in the suspension, *V* is the volume of the sample.

The following dimensionless quantities are introduced in Equation 3–Equation 6:

[7]
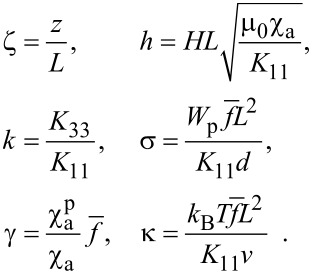


Here ζ is the dimensionless coordinate, *k* is the ratio of the Frank constants, σ is the dimensionless anchoring energy of the CNTs on the NLC matrix, *h* is the dimensionless magnetic field strength. The unit of the field strength is 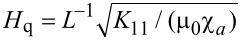, which, up to the factor π, corresponds to the Fréedericksz transition field in a pure NLC [31].

The parameter γ characterizes the relative contribution of the influence of a magnetic field on the orientational structure of the liquid crystal suspension. For γ ≫ 1 the deformation of the orientational structure of the suspension is due to the presence of the impurity CNTs, and for γ ≪ 1 the appearance of distortions of the NLC director is due to the diamagnetic nature of the NLC matrix.

The expression in Equation 5 for the reduced volume fraction *g*(ζ) of CNTs containing the parameter κ describes the spatial redistribution of the CNTs inside the layer under the action of a uniform magnetic field. Diamagnetic nanotubes will migrate to the part of the layer where the sum of their magnetic and orientational energies in the NLC matrix is minimal. The analogue of this effect in the physics of ferronematics and ferrocholesterics (magnetic suspensions of ferromagnetic nanoparticles in LCs) is called the magnetic segregation effect [12]. It has a significant influence on the type of orientation transitions in LC composite materials [40–44], and, as predicted in [39], must appear in the CNT suspensions on the basis of an LC.

The segregation parameter κ is responsible for the intensity of the concentration stratification of CNTs in an LC. With κ ≫ 1, the redistribution of the CNTs in an LC layer can be neglected, considering the volume fraction of CNTs to be constant inside the layer. At κ ≤ 1 the concentration stratification becomes substantial.

Let us estimate the dimensionless parameters in Equation 7. According to [45] for a typical NLC the anisotropy of the diamagnetic susceptibility χ_a_ ≈ 10^−6^, and the elastic constants *K*_33_, *K*_11_ ≈ 10^−12^ N. The anisotropy of the diamagnetic susceptibility of CNTs [14,20–22] reaches values of 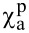 ≈ 10^−5^ to 10^−4^. Assuming the average volume fraction of the CNTs 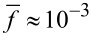
[9], the temperature *T* = 300 K, the layer thickness *L* = 40 μm, we get *k* ≈ 1, γ ≈ 10^−2^ to 10^−1^, κ ≈ 10 for small CNTs (*l* ≈ 2 μm, *d* ≈ 20 nm) and κ ≈ 10^−1^ for large CNTs (*l* ≈ 10 μm, *d* ≈ 50 nm). The value of the parameter κ in the first case corresponds to weak segregation, and in the second case to strong segregation. The anchoring energy between the LC molecules and the CNTs can vary over a wide range of *W*_p_ ≈ 10^−7^ to 10^−3^ N/m [33–34,37,46], which gives σ ≈ 1 to 10^4^.

Equation 3–Equation 6, under the conditions of rigid planar anchoring of the NLC director **n** on the boundaries of the layer

[8]
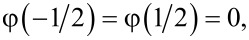


allow us to determine the equilibrium spatial distributions of the volume fraction of CNTs *g*(ζ), and the angles φ(ζ) and ψ(ζ) of the orientations of the NLC and CNTs directors.

Note that due to the symmetry of the boundary conditions (Equation 8), the maximum deviation of the director from the axis of easy orientation is achieved in the middle of the layer:

[9]
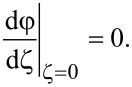


Next, we integrate the system of the integro-differential Equation 3–Equation 6. To do this, we multiply Equation 3 by dφ/dζ, and Equation 4 by *g*dψ/dζ and sum them up. After integrating this equation taking into account the condition given in Equation 9, we obtain

[10]
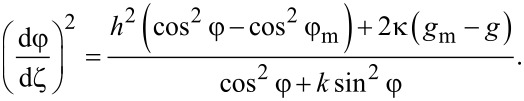


where φ_m_ = φ(0), ψ_m_ = ψ(0) are the angles of orientation of the NLC and CNT directors in the center of the layer, and *g*_m_ = *g*(φ_m_, ψ_m_) is the reduced volume fraction of CNTs in the center of the layer.

The integration of Equation 10 over ζ *>* 0 with the boundary conditions in Equation 8 gives the following equation:

[11]
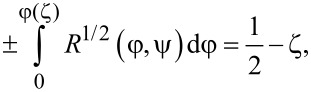


where

[12]
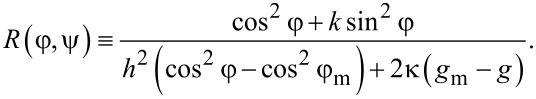


Equation 11 determines the implicit dependence of the angle φ(ζ) of the orientation of the NLC director on the coordinate in the upper half of the layer, i.e., for ζ ∈ [0,1/2]. The plus sign in Equation 11 corresponds to the solutions that describe the counter-clockwise rotation of the NLC director (φ > 0). Hereafter, we will consider these particular solutions. The minus sign in Equation 11 corresponds to the clockwise rotation of the NLC director (φ *<* 0).

In the middle of the layer, where the director rotation angle φ(0) = φ_m_ reaches the maximum value, Equation 11 takes the form

[13]
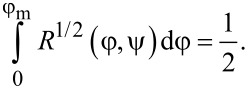


Having transformed the normalization integral *Q* in Equation 6 using the relation in Equation 10, for the CNT distribution function *g*(ζ) we get the following integral equation:

[14]
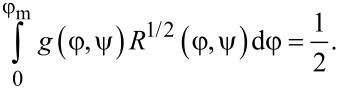


Equation 11–Equation 14 together with coupling Equation 4 allows us to determine the angles φ(ζ) and ψ(ζ), as well as the distribution function of the CNTs *g*(ζ) in the layer, depending on the applied magnetic field *h* and the parameters of the liquid crystal composite *k*, σ, γ and κ.

## Results and Discussion

### Fréedericksz transition and critical fields

The orientational equilibrium Equation 3–Equation 6 have two solutions that correspond to the homogeneous distribution of the CNTs (*g* = 1) and the uniform fields **n** and **m**. One of the solutions φ(ζ) = ψ(ζ) = 0 corresponds to the initial planar phase of the NLC–CNT mixture with a parallel orientation of the directors of the NLC and the CNTs (**n**∥**m**⟂**H**). Another uniform solution φ(ζ) = 0, ψ(ζ) = π/2 corresponds to the homeotropic phase with mutually orthogonal orientations of the directors (**n**⟂**m**∥**H**). Nonuniform solutions of Equation 3–Equation 6 describe a nonuniform angular phase of the NLC–CNT mixture, in which the angle between the directors **n** and **m** is not zero and π/2.

Linearization of Equation 3–Equation 6 close to uniform solutions lets us obtain analytical expressions for the critical fields of transitions between the planar, angular, and homeotropic phases [39]:

[15]
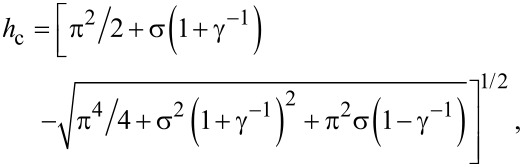


[16]
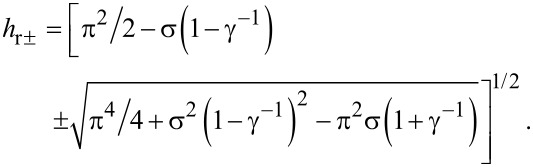


The field *h*_c_ in Equation 15 corresponds to the Fréedericksz transition field from the initial uniform planar phase to the nonuniform angular one. Equation 16 defines two threshold fields *h*_r±_, where *h*_r+_ corresponds to the transition from the angular phase to the homeotropic one, and *h*_r−_ characterizes the subsequent transition from the homeotropic phase to angular one.

**Figure 2:**
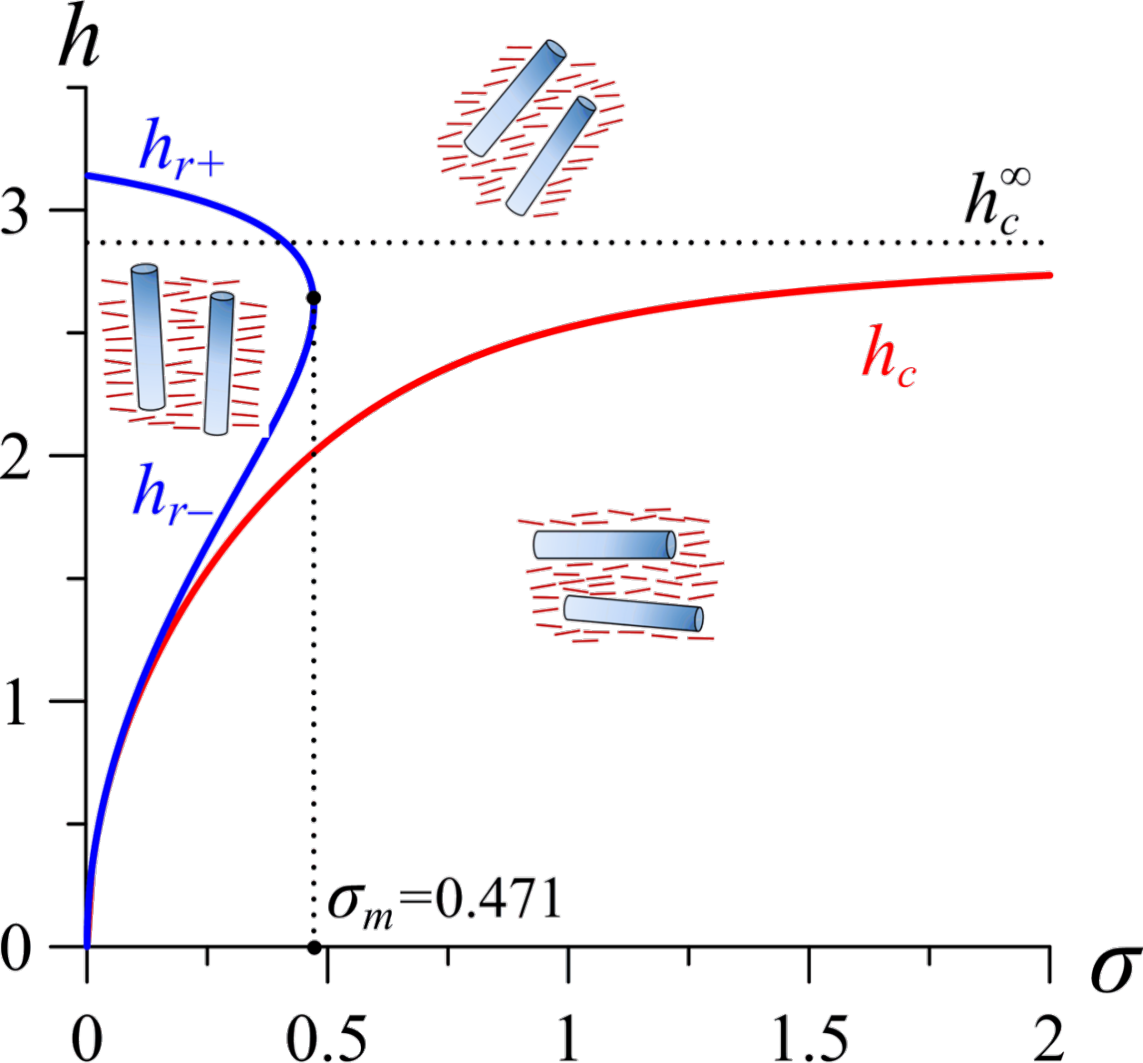
Diagram of orientation phases of the NLC–CNT mixture for γ = 0.2. The horizontal dotted line corresponds to the Fréedericksz field 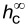 = 2.868 under the condition of rigid anchoring between the subsystems.

The diagram of the orientation phases of the NLC–CNT mixture, plotted using Equation 15 and Equation 16, is shown in Figure 2. The region under the curve *h*_c_(σ) corresponds to the planar phase (**n**∥**m**⟂**H**). The region to the left of the curves *h*_r+_(σ) and *h*_r−_(σ) corresponds to the homeotropic phase (**n**⟂**m**∥**H**). The region above the curve *h*_c_(σ) corresponds to the angular phase with nonuniform directors **n** and **m** inside the layer.

Analysis of the solutions of Equation 16 shows that the homeotropic phase can exist only under the condition of weak anchoring between the NLC matrix and the CNTs for σ ≤ σ_m_, where

[17]
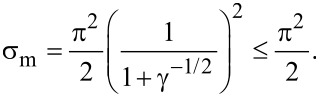


According to the molecular statistical theory [47], for highly anisometric impurity particles, such as carbon nanotubes, the anchoring between the CNTs and the NLC matrix should be sufficiently strong. Thus, for real NLC–CNT suspensions, the condition σ *>* σ_m_ is fulfilled and an orientational transition from the planar to the angular phase occurs in the system, which is consistent with the experimental works [27–28,48]. The above estimates of dimensionless quantities (Equation 7) show that the parameter σ takes large values, which corresponds to sufficiently strong anchoring of the CNTs and the NLC matrix. Under the condition of absolutely rigid anchoring (σ→∞), Equation 15 is considerably simplified:

[18]
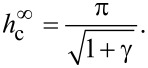


Recently [39], first-order and second-order phase transitions between orientation states of NLC–CNT mixture have been predicted. For the Fréedericksz transition from the planar to the angular phase, the value of the segregation parameter κ_*_ corresponding to the tricritical point is determined by the following expression [39]:

[19]
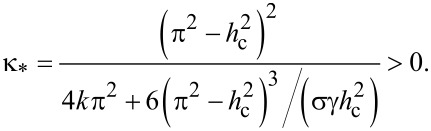


With κ ≥ κ_*_, the Fréedericksz transition corresponds to the second-order phase transition, and with κ *<* κ_*_ to the first-order phase transition. In the case of absolutely rigid anchoring of CNTs with the NLC molecules, Equation 19 takes the form

[20]
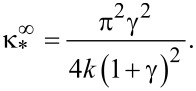


Note that (Equation 15) allows us to determine only the fields of the second-order orientational transitions. Let us find the field *h*_e_ of the first-order equilibrium Fréedericksz transition. The free energy *F* (Equation 1) of the NLC–CNT layer in a dimensionless form will be written as follows:

[21]
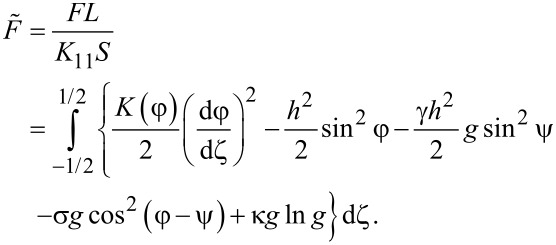


The field of the equilibrium transition of the first order *h*_e_ can be found from the equality of the free energies of the planar and angular phases. Passing in Equation 21 from integration over the coordinate ζ to integration over the angle φ using Equation 10, the condition of equality of the free energies of the phases is written as follows:

[22]
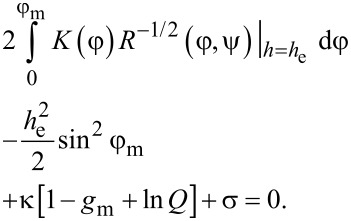


Equation 22, together with Equation 4, Equation 13 and Equation 14, allows us to find the field of the first-order equilibrium transition *h*_e_ between the planar and angular phases of the NLC–CNT mixture.

### Orientational and concentration distributions

**Figure 3:**
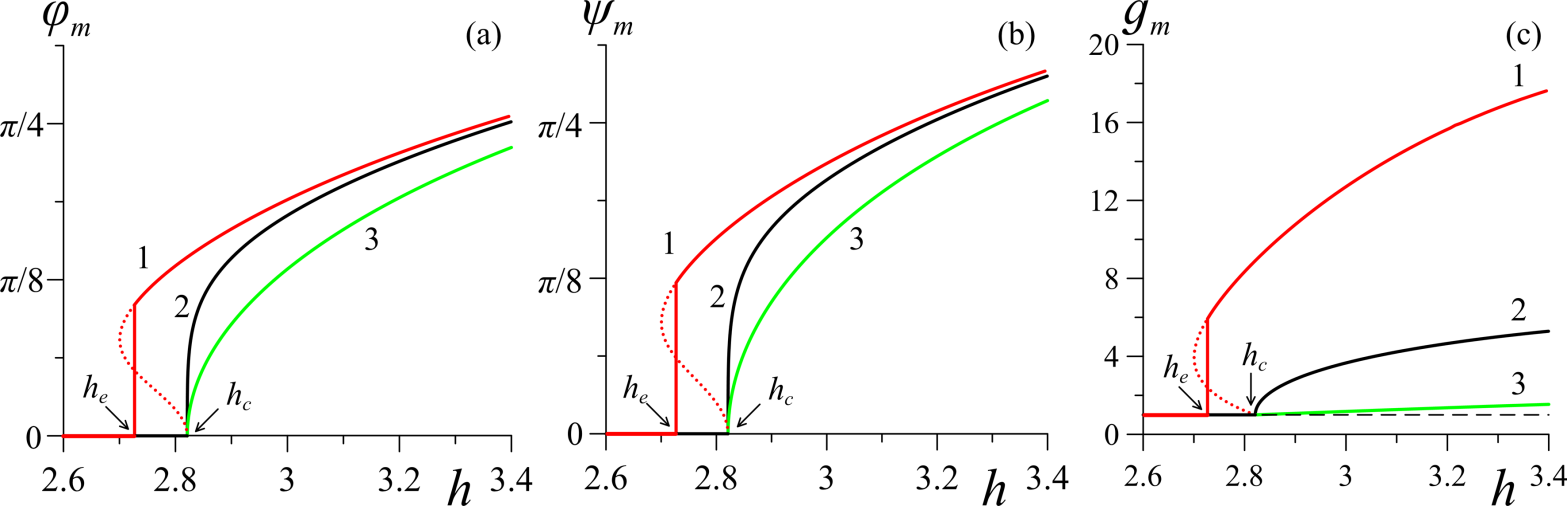
Dependencies of (a) the angle φ_m_, (b) the angle ψ_m_, and (c) the reduced CNT volume fraction *g*_m_ in the center of the layer on the applied magnetic field *h* for γ = 0.2 and σ = 5: curve 1 – κ = 0.01; curve 2 – κ = κ_*_ = 0.056; curve 3 – κ = 0.5. Here *h*_c_ = 2.824, *h*_e_ = 2.727.

The results of the numerical solution of Equation 4 and Equation 11–Equation 14 are presented in Figure 3–Figure 6 (see below). Figure 3 shows the orientation angles φ_m_, ψ_m_ as functions of the magnetic field strength and the reduced CNT volume fraction *g*_m_ in the center of the layer. The solid lines in the figure correspond to thermodynamically stable states, the dotted lines correspond to metastable states, and the vertical segments of the straight lines show the first-order phase transition. The calculations were carried out for σ = 5, γ = 0.2, *k* = 1.5 and various values of the segregation parameter κ. The orientation phase diagram for the selected parameters is shown in Figure 2. The value of the second-order transition field *h*_c_ = 2.824 from the planar phase into the angular phase was found using Equation 15, the value of the tricritical segregation parameter κ_*_ = 0.056 was determined using Equation 19. In the limit of absolutely rigid anchoring for these parameter values, using Equation 18 and Equation 20, respectively, we obtain the Freedericksz transition field 
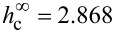
 and the segregation parameter corresponding to the tricritical point 
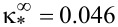
.

As noted above, for κ ≥ κ_*_, transitions from the initial planar phase to the angular phase are transitions of the second order. In this case, distortions of the orientational structure of the suspension appear continuously when the magnetic field strength reaches *h*_c_ = 2.824, above which the directors of NLC and CNTs begin to orient in the direction of the field, which corresponds to an increase in the angles φ_m_(*h*) and ψ_m_(*h*) (curves 2 and 3 in Figure 3a,b). In addition, with increasing distortions in the orientational structure of the NLC director, there is an increase in the concentration of CNTs in the center of the layer due to the segregation effect (see Figure 3c). When κ *<* κ_*_, the Fréedericksz transition in suspension is a first-order transition, which corresponds to the vertical segments of curves 1 in Figure 3. Note that the field of the equilibrium transition of the first order, *h*_e_ = 2.727, is smaller than the field *h*_c_ = 2.824 from Equation 15. As the field grows, the angles φ_m_ and ψ_m_ approach the value of *π/2*, i.e., the directors **n** and **m** of the NLC and CNTs are oriented in the direction of the field **H**.

**Figure 4:**
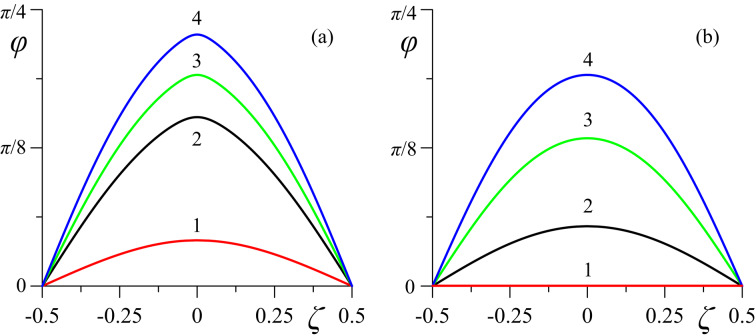
Spatial distributions of the orientation angle φ(ζ) of the NLC director **n** for different values of the magnetic field strength *h* in the case of (a) strong (κ = 0.01) and (b) weak (κ = 0.5) segregation of impurity CNTs: curve 1 – *h* = 2.75; curve 2 – *h=2.85*; curve 3 – *h* = 3.0; curve 4 – *h* = 3.2.

**Figure 5:**
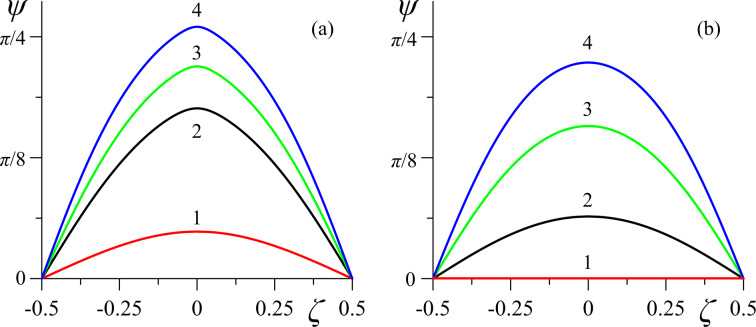
Spatial distributions of the orientation angle ψ(ζ) of the CNT director **m** for different values of the magnetic field strength *h* in the case of (a) strong (κ = 0.01) and (b) weak (κ = 0.5) segregation of impurity CNTs: curve 1 – *h* = 2.75; curve 2 – *h* = 2.85; curve 3 – *h* = 3.0; curve 4 – *h* = 3.2.

**Figure 6:**
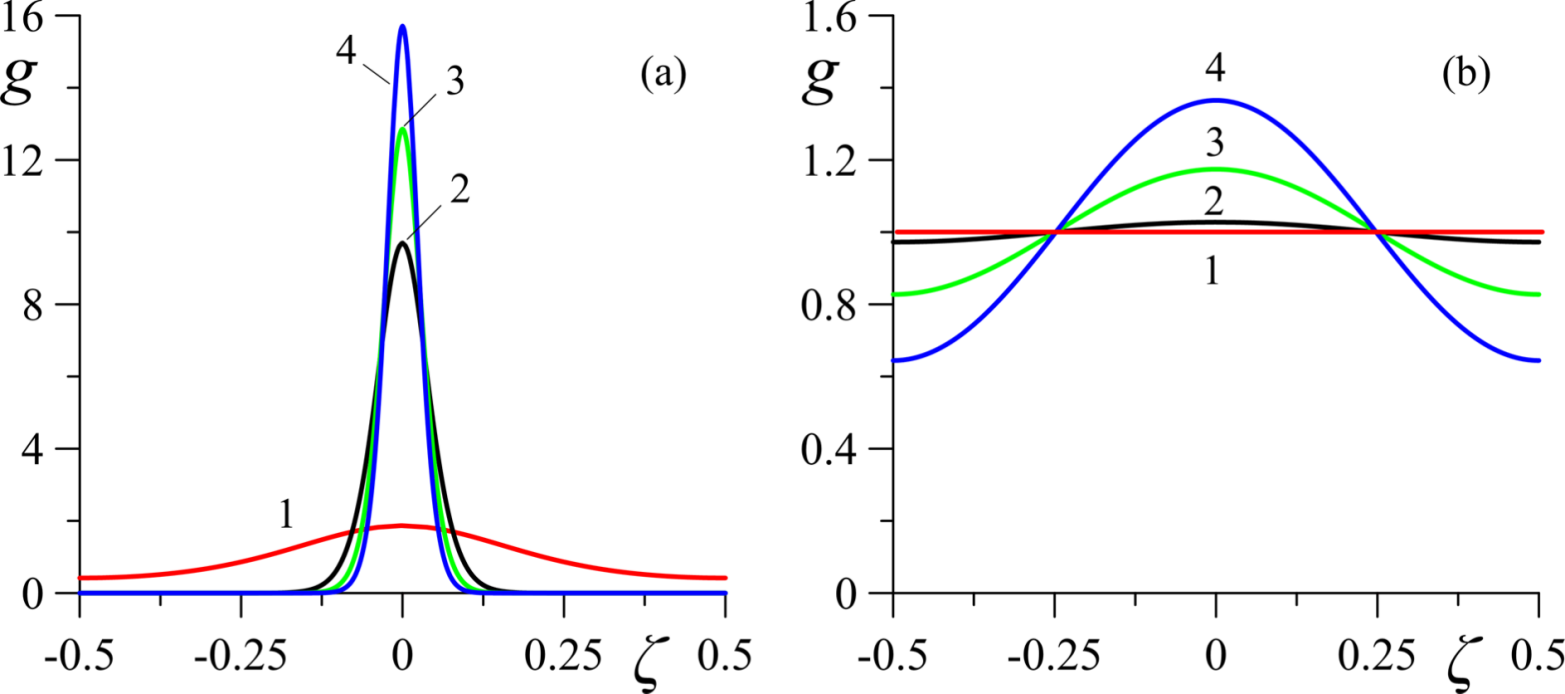
Spatial distributions of the reduced CNT volume fraction *g* for different values of the magnetic field strength *h* in the case of (a) strong (κ = 0.01) and (b) weak (κ = 0.5) segregation of CNTs: curve 1 – *h* = 2.75; curve 2 – *h* = 2.85; curve 3 – *h* = 3.0; curve 4 – *h* = 3.2.

Figure 4–Figure 6 present the spatial distributions of the orientation angles φ(ζ) and ψ(ζ) of the directors and the reduced volume fraction *g*(ζ) of CNTs for different values of the magnetic field strength *h*. The cases of strong (κ = 0.01 in Figure 4a, Figure 5a, Figure 6a) and weak (κ = 0.5 in Figure 4b, Figure 5b, Figure 6b) segregation of CNTs in the layer have been examined.

At *h* = 2.75 (*h*_e_ *< h < h*_c_) in case of strong segregation in the suspension, there are significant distortions of the orientational structure (curves 1 in Figure 4a and Figure 5a), while in the case of weak segregation, the suspension is in the uniform planar phase (curves 1 in Figure 4b and Figure 5b). For the magnetic field *h* = 2.85 *> h*_c_, the distortions of the orientational structure become even greater with strong segregation (curves 2 in Figure 4a and Figure 5a) and appear with weak segregation of CNTs (curves 2 in Figure 4b and Figure 5b). As the field increases, the orientation angles of the directors of the NLC, φ(ζ), and CNTs, ψ(ζ), grow in the whole layer (curves 3 and 4 in Figure 4 and Figure 5).

From Figure 6 it can be seen that as a result of segregation at κ = 0.01 an increase in the magnetic field leads to the accumulation of CNTs in the middle of the layer, leaving the boundary regions of the layer to be depleted in CNTs. At κ = 0.5 (Figure 6b), the redistribution of CNTs with increasing field is not so significant. Interestingly, in this case, the CNT volume fraction *g*(ζ) ≈ 1 at two spatial points ζ = ±1/4 does not change with increasing field and remains close to the value corresponding to the homogeneous planar phase.

In the case of sufficiently strong anchoring (σ = 5) between the directors **n** and **m**, the distribution of φ(ζ) does not qualitatively differ from ψ(ζ), as can be seen from the comparison in Figure 4 and Figure 5. Qualitative differences in the behavior of φ(ζ) and ψ(ζ) should be expected at σ ≃ σ_m_, where the appearance of the homeotropic phase is not prohibited, which can lead to reentrant orientational transitions. For example, in ferronematics (suspensions of anisometric ferromagnetic particles based on NLCs [12]), analogous transitions were studied in [43–44,49].

### Magneto-optical response of the NLC–CNT mixture

One of the ways to study orientational transitions in an LC experimentally is to measure birefringence in a cell. In an optically positive LC, with the appearance of distortions in the orientational structure, the refractive index of the ordinary ray *n*_o_ remains unchanged, and the refractive index of the extraordinary ray *n*_e_ will decrease and approach the value of *n*_o_. Above the Fréedericksz transition the value of the effective refractive index *n*_eff_ depending on the angle of the director orientation is determined by the following relation [45]:

[23]
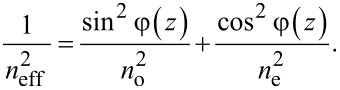


The optical phase lag δ between ordinary and extraordinary rays of monochromatic light with a wavelength of λ passing through a cell can be determined by integrating *n*_eff_ − *n*_o_ over the thickness of the layer *L*[45]:

[24]
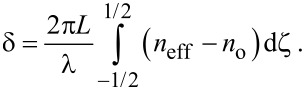


To calculate the optical phase lag, in Equation 24 we change from integration over the coordinate ζ to integration over the angle φ using Equation 10, then Equation 24 can be written as

[25]
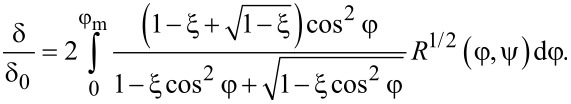


Here, δ_0_ = 2π*L*(*n*_e_ − *n*_o_)/λ is the phase lag in the absence of a magnetic field, and the notation 
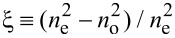
 is introduced. In Equation 25, the angles φ_m_ and ψ_m_, and the distribution function *g* are given by Equation 4, Equation 13 and Equation 14, and the function *R*(φ,ψ) by Equation 12.

**Figure 7:**
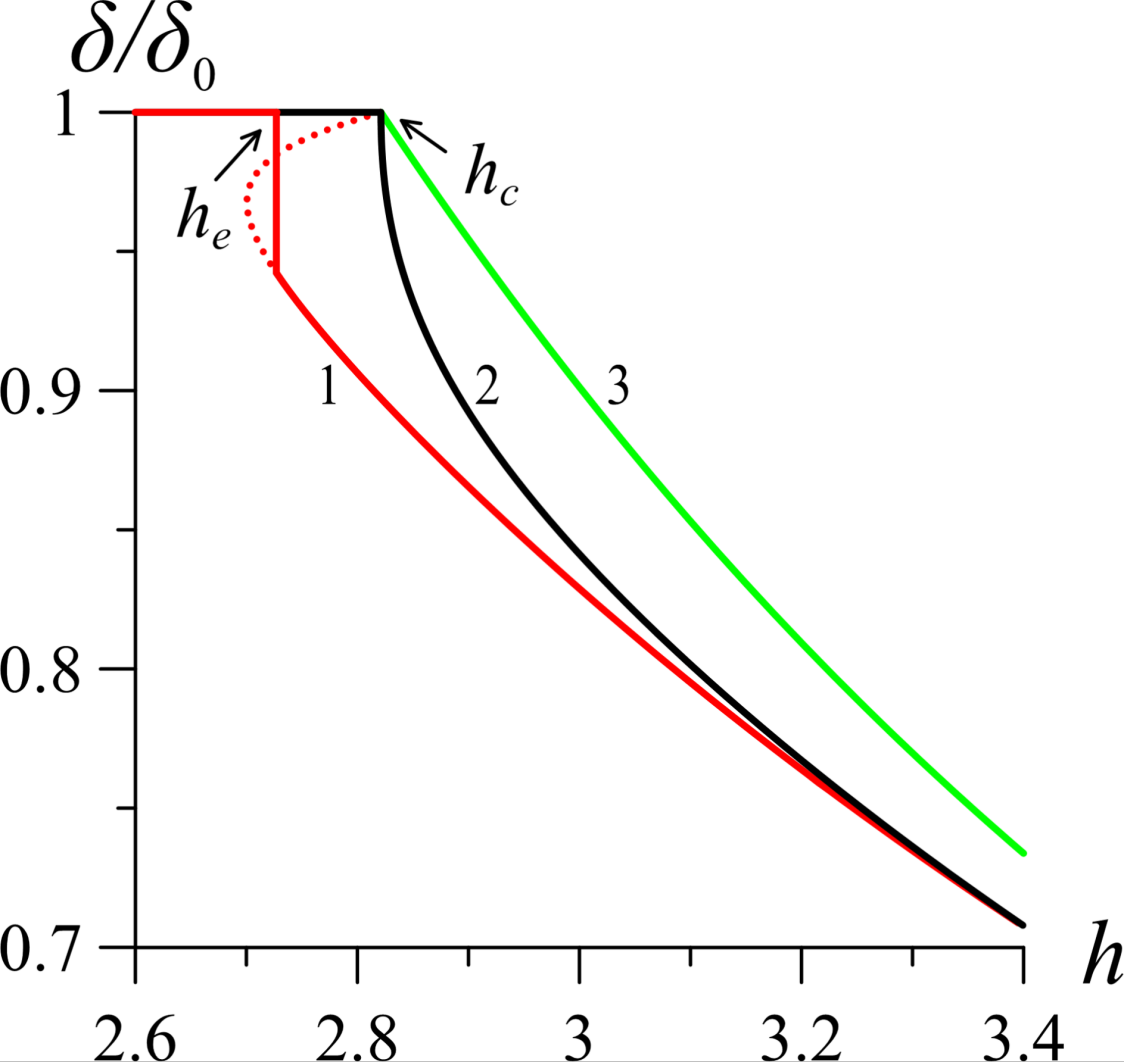
Dependence of the optical phase lag of the suspension on the applied magnetic field *h* for γ = 0.2 and σ = 5: curve 1 – κ = 0.01; curve 2 – κ = κ_*_ = 0.056; curve 3 – κ = 0.5. Here *h*_c_ = 2.824, *h*_e_ = 2.727.

Figure 7 presents the results of calculations of the reduced phase lag (Equation 25) between the ordinary and extraordinary rays of monochromatic light passing through the NLC cell with a CNT impurity. For calculations we use the values of the refractive index of the liquid crystal 5CB (*n*_o_ = 1.53, *n*_e_ = 1.71 for λ = 632.8 nm [45]), on the basis of which NLC–CNT mixtures can be prepared [24,32,50]. From Figure 7 it can be seen that in the initial planar phase the reduced phase lag is maximum and corresponds to one. As the magnetic field increases, distortions of the orientational structure appear (Figure 3), and the phase lag begins to decrease. In the case of strong segregation of CNTs (curve 1 in Figure 7), the optical phase lag changes abruptly, which corresponds to the first-order phase transition. In the case of weak segregation (curves 2 and 3 in Figure 7), distortions in the orientation structure of NLC–CNT mixture appear continuously and increase with increasing magnetic field.

## Conclusion

We have studied the influence of magnetic segregation effects on the orientation structure and the magneto-optical response of CNT suspension in an NLC. A flat layer of the NLC–CNT suspension in a uniform magnetic field has been considered. The anchoring between the CNT and NLC subsystems was assumed to be finite and planar.

We have performed numerical calculations of the rotation angles of the NLC and CNT directors inside the layer. The orientational and concentration distributions in the layer have been obtained for various values of the magnetic field strength and the segregation parameter. It has been shown that the Fréedericksz transition in an NLC doped with CNTs can be a transition of the first or the second order depending on the degree of segregation. In the case of weak magnetic segregation, the orientational transition is a second-order phase transition, like the Fréedericksz transition for pure LCs. At strong segregation, the Fréedericksz transition becomes a first-order transition, causing the bistable behavior of the NLC and CNTs directors. We have obtained the field of the first-order equilibrium transition between the planar and angular phases of the suspension. The optical phase lag between ordinary and extraordinary rays after their passing through the LC layer has been calculated as a function of the strength of the applied magnetic field.

From the obtained results it follows that it is possible to experimentally observe the Fréedericksz transition, corresponding to the first-order phase transition, only in LC doped with sufficiently large carbon nanotubes (*l* ≈ 10 μm, *d* ≈ 50 nm, 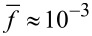). In addition, the CNTs must have a high anisotropy of the diamagnetic susceptibility (γ ≥ 0.1). For a rough estimate of the tricritical value of the segregation parameter κ_*_, one can use Equation 20, which is valid for rigid anchoring between CNTs and the LC matrix.
